# Experimental evidence for UNC-6 (netrin) axon guidance by stochastic fluctuations of intracellular UNC-40 (DCC) outgrowth activity

**DOI:** 10.1242/bio.20136346

**Published:** 2013-10-23

**Authors:** Gauri Kulkarni, Zhennan Xu, Ahmed M. Mohamed, Haichang Li, Xia Tang, Gerard Limerick, William G. Wadsworth

**Affiliations:** Department of Pathology, Robert Wood Johnson Medical School, Rutgers University, 675 Hoes Lane West, Piscataway, NJ 08854, USA

**Keywords:** *Caenorhabditis elegans*, DCC receptor, Netrin, Stochastic Process, UNC-6, Axon guidance, Wnt signaling, Neurons

## Abstract

How the direction of axon guidance is determined is not understood. In *Caenorhabditis elegans* the UNC-40 (DCC) receptor mediates a response to the UNC-6 (netrin) guidance cue that directs HSN axon development. UNC-40 becomes asymmetrically localized within the HSN neuron to the site of axon outgrowth. Here we provide experimental evidence that the direction of guidance can be explained by the stochastic fluctuations of UNC-40 asymmetric outgrowth activity. We find that the UNC-5 (UNC5) receptor and the cytoskeletal binding protein UNC-53 (NAV2) regulate the induction of UNC-40 localization by UNC-6. If UNC-40 localization is induced without UNC-6 by using an *unc-53* mutation, the direction of UNC-40 localization undergoes random fluctuations. Random walk models describe the path made by a succession of randomly directed movement. This model was experimentally tested using mutations that affect Wnt/PCP signaling. These mutations inhibit UNC-40 localization in the anterior and posterior directions. As the axon forms in Wnt/PCP mutants, the direction of UNC-40 localization randomly fluctuates; it can localize in either the anterior, posterior, or ventral direction. Consistent with a biased random walk, over time the axon will develop ventrally in response to UNC-6, even though at a discrete time UNC-40 localization and outgrowth can be observed anterior or posterior. Also, axon formation is slower in the mutants than in wild-type animals. This is also consistent with a random walk since this model predicts that the mean square displacement (msd) will increase only linearly with time, whereas the msd increases quadratically with time for straight-line motion.

## Introduction

The HSN neuron of *Caenorhabditis elegans* extends short neurites in different directions immediately after the animal hatches. These neurites, which dynamically extend and retract filopodia, become restricted to the ventral side of the neuron where a leading edge forms. Multiple neurites extend from this surface until one develops into a single axon extending to the ventral nerve cord (Adler et al., 2006). Axon formation is easily observed and the asymmetric localization of proteins during this process can be studied (Adler et al., 2006; Garriga et al., 1993). During axon formation, extracellular guidance cues polarize protrusive activity within HSN (Adler et al., 2006; Quinn et al., 2006). In particular, the UNC-6 (netrin) guidance cue promotes ventral axon formation by causing the UNC-40 (DCC) receptor to localization to the ventral side of the neuron (Adler et al., 2006). It's been shown that the ability of UNC-40 to localize to one side of the neuron is controlled by the molecular properties of UNC-6-ligated UNC-40 (Xu et al., 2009).

It is commonly understood that the process that determines the direction of HSN axon guidance is deterministic (Tessier-Lavigne and Goodman, 1996). In a deterministic system no randomness is involved in the development of future states of the system. Through molecular mechanisms encoded by the neuron, the interaction between the HSN neuron and the UNC-6 guidance cue causes an attractive response. The attractive response instructs the cytoskeleton to produce an internal force directed towards the side of the neuron nearest the UNC-6 source. Therefore, the neuron's attractive response to the cue determines the direction of axon guidance.

In this study we ask whether the direction of UNC-6 guidance might be determined via a stochastic process rather than a deterministic one. A stochastic process evolves in time via random changes. If the response to UNC-6 occurs through a stochastic process the direction of axon outgrowth would be randomly determined. A random walk is mathematical model that describes the path of an object as a succession of steps in which the direction of each move is randomly determined. The development of random walk theory can be traced back to the study of the movement of pollen particles made famous by Brown (Brown, 1828). Einstein's model of Brownian motion in known as a random walk formalization (Einstein, 1906; Einstein, 1956). Random walks are used to explain the behaviors of the movements of particles in liquids and gases, forging animals, and stock prices. A biased random walk describes a path that results from a consistent bias in a preferred direction. Biased random walks have been used to model how cells such as *Dictyostelium*, yeast, and bacteria can follow extracellular chemical gradients (Arrieumerlou and Meyer, 2005; Dyer et al., 2013; Keller and Segel, 1971; Macnab and Koshland, 1972; van Haastert and Postma, 2007).

Experimental evidence for a biased random walk is obtained by asking whether the system behaves as a random walk when it is manipulated. In this paper we observe the behavior of the system by removing the influence of different external asymmetric guidance cues that regulate the probability of movement in different directions. Previously we reported evidence that the HSN response to UNC-6 is indeterminate for the direction of UNC-40 localization and axon outgrowth. An UNC-40 variant, UNC-40 (A1056V), induces an asymmetrical localization of UNC-40 in HSN even in the absence of UNC-6 (Xu et al., 2009). UNC-40 localizes to any one side of the neuron and the direction of axon outgrowth is arbitrary. It is proposed that the conformation of UNC-40 (A1056V) mimics the UNC-6-ligated UNC-40 confirmation. We now show that the cytoskeletal binding protein UNC-53 (NAV2) and the UNC-5 (UNC5) receptor regulate the induction of UNC-40 asymmetric localization. Further, in double *unc-53;unc-6* mutants, the asymmetric localization of wild-type UNC-40 is induced but the direction of localization is indeterminate. Direct observations show that UNC-40 has a probability of being present at each side of the neuron during the formation of the single leading edge of axon outgrowth. This experimentally provides evidence that the asymmetric localization of UNC-40 occurs through a random process. The extracellular distribution of UNC-6 has a major influence on the direction of HSN axon formation. In wild-type animals, random fluctuations are not observed; UNC-40 localizes ventrally. However, we find that EGL-20 (Wnt), the Wnt receptor MIG-1 (Frizzled), the planar cell polarity (PCP) component VANG-1 (Van Gogh), or the UNC-40 effectors, MIG-10 (Lamellipodin) and MADD-2 (TRIM) inhibit intracellular UNC-40 localization in the anterior or posterior directions. In the mutants the probability of UNC-40 being present at a specific side of the neuron is altered; UNC-40 localizes with some probability to either the ventral, anterior, or posterior directions during initial axon formation. The probability of UNC-40 localization in the ventral direction is higher than the probability of anterior or posterior localization. This allows a biased random walk model to be experimentally tested. We observe that the behavior of the system approximates a biased random walk. As predicted, even though the direction of UNC-40 localization can be in the anterior or posterior direction, axon formation will nevertheless be guided towards the ventral source of UNC-6 over time. This is consistent with the direction of guidance being determined by the sequence of stochastic fluctuations of UNC-40-mediated axon outgrowth activity, rather than by the direction of UNC-40 localization and axon outgrowth at any discrete time. We also observe that the time in which it takes for an axon to form is delayed in the mutants. This is consistent with how displacement is described by a random walk. That is, the mean square displacement will tend to increase only linearly with time, not quadratically with time as is the case of straight-line motion. Together these results indicate that the direction and timing of UNC-6 guidance can be explained by the stochastic fluctuation of intracellular UNC-40 outgrowth activity and the exertion of a bias by multiple cues.

## Materials and Methods

### Strains

All strains were maintained as described (Brenner, 1974). Strains were grown at 20°C unless stated otherwise. In addition to the wild-type *C. elegans* variety Bristol strain N2, strains containing the following mutations and transgenes were used in these studies:

LGI, *mig-1(e1787), unc-40(e1430), unc-40(e1430, ur304)*; LGII *unc-53(n152)* LGIII, *mig-10(ct41)*; LGIV, *unc-5(e53)*, *egl-20(n585), kyIs262 (unc-86::myr-gfp, odr-1::dsRed)*; LGV, *rpm-1(ur299), madd-2 (ky592)*; LGX, *unc-6(ev400)*, *vang-1(tm1422)*. Transgenes maintained as extrachromosomal arrays included: *zdIs5 [mec-4::GFP]*, *kyEx1212 (unc-86::unc-40::gfp, odr-1:: dsRed), kyEx927 (unc-86::mig-10::gfp, odr-1:: dsRed)* [Provided by Dr Cori Bargmann, Rockefeller University, USA], *gmEx384(unc-86::mig-1::gfp, dpy-30::NLS::DsRed)* [Provided by Dr Gian Garriga, University of California, Berkeley]. Some strains were provided by the Caenorhabditis Genetics Center.

Genotypes of strains carrying multiple mutations were confirmed by PCR for deletions or sequencing of individual mutations. All mutant alleles used in this study either eliminate or severely reduce gene function. The hermaphrodite sex was analyzed in all experiments.

### Analysis of phenotypes

For analysis of the AVM axon protrusion phenotype, L4 stage larvae were mounted on a 5% agarose pad. The AVM axon was visualized in L4 stage larvae expressing the *zdIs5* transgene, which encodes *mec-4*::GFP. An anterior protrusion was scored as the axon traveling laterally more than 3 cell body length from the cell body. Dorsal or posterior protrusion was scored as the axon traveling dorsally or posteriorly for a distance greater than two cell body lengths from the cell body. The AVM was considered multipolar if more than one process, greater than one cell body length, was observed.

HSN axon morphology was visualized using an integrated *unc-86::myrGFP* transgenic strain, *KyIs262*. Synchronized populations were obtained by allowing eggs to hatch overnight in M9 buffer without food. The resulting L1 nematodes were fed and grown at 20°C to the specified developmental stage. The HSN development at different larval stages in mutants (L1–L4) was followed by the gonad size, (early and late L2, early L3), distal tip cell migration (mid-L3) and P6.p cell invagination (L3–L4 transition and mid-L4) as markers (Adler et al., 2006). An anterior protrusion was scored as the axon traveling laterally for a distance greater than three cell bodies from the cell body. Dorsal or posterior protrusion was scored as the axon traveling dorsally or posteriorly for a distance greater than two cell body lengths from the cell body. The HSN was considered multipolar if more than one process, greater than one cell body length, was observed.

### Image analysis

For analysis of UNC-40::GFP in HSN neurons, L2 stage larvae expressing the *kyEx1212[ unc-86::unc-40::GFP; odr-1::dsRed]* transgene were mounted in M9 buffer with 10 mM levamasole. Images were taken using epifluorescent microscopy with a Zeiss 63× water immersion objective. To judge the HSN dorsoventral position and developmental stage, each larva was also imaged by differential interference contrast (DIC) microscopy. The UNC-40::GFP localization was determined by measuring the average intensity under lines drawn along the dorsal and ventral edges of each HSN cell body by using IP lab software.

For analysis of the anterior–posterior orientation of UNC-40::GFP along the dorsal surface, the dorsal segment was geometrically divided into three equal lengths (dorsal anterior, dorsal central and dorsal posterior segments). The line-scan intensity plots of each of these segments were recorded. ANOVA test was used to determine if there is a significant difference between intensities of three segments. The dorsal distribution was considered uniform if *P*≧0.05 and was considered asymmetrical if *P*≤0.05. Further, within an asymmetric population, the highest percent intensity was considered to localize UNC-40::GFP to either anterior, posterior or central domain of the dorsal surface.

### Assay for independence of HSN cell migration and axon outgrowth

For Table 3, the phenotypes were scored regardless of the position of cell body. However, the pattern of axon migration appears to be independent of the cell body position. Independence of HSN cell migration and axon outgrowth defects in *egl-20* and *mig-1* mutants were determined by Chi-square (χ^2^) analysis. In this assay, only HSNR migration and axon outgrowth was quantified. The HSNR cell migration in *egl-20* and *mig-1* mutants was considered as defective if it migrated less than 70% of the distance between anus and vulva (Garriga et al., 1993). The genotypes were analyzed with one degree of freedom: *egl-20(n585)*, χ^2^ = 0.014, *P* = 0.90; *mig-1(n1787)* χ^2^ = 1.346, *P* = 0.24; these *P* values (>0.05) indicate that HSN cell migration and HSN axon defects are independent events.

## Results

### Genetic interaction between *unc-53* and *unc-6*

UNC-53 (NAV2) is a cytoskeletal binding protein related to the mammalian Neuronal Navigators (NAVs) and in *unc-53* mutants axon extension and guidance are defective (Hekimi and Kershaw, 1993; Schmidt et al., 2009; Stringham et al., 2002; Stringham and Schmidt, 2009). The NAV proteins are proposed to modulate cytoskeletal dynamics at the leading edge of cells and could have specialized roles in receptor trafficking (Stringham and Schmidt, 2009). In *C. elegans*, the *unc-53* gene functions cell autonomously in the migration of cells and axons (Stringham et al., 2002).

**Fig. 1.**
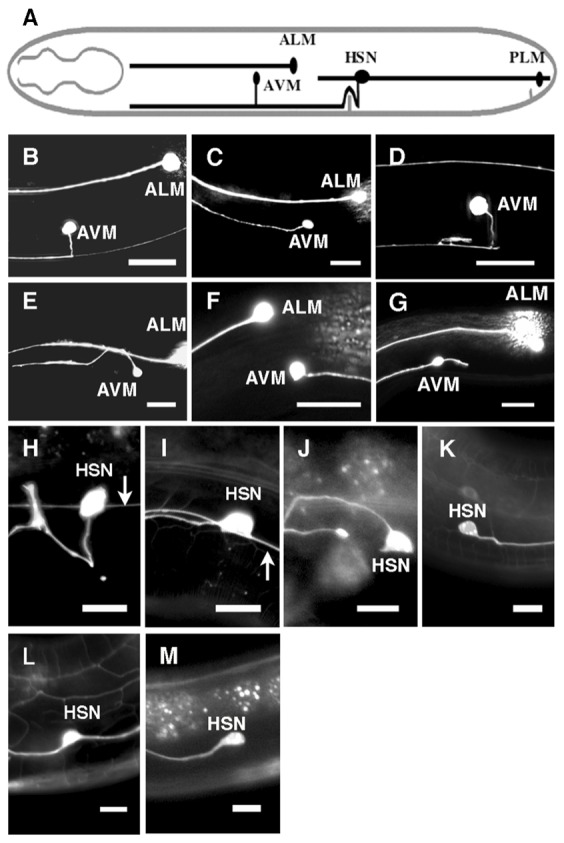
Genetic interactions between *unc-53* and *unc-6* and between *unc-53* and *unc-5* affect the direction of axon protrusion. (A) Schematic diagram of AVM and HSN axon outgrowth. Axon outgrowth is towards ventral UNC-6 sources. (B–G) Photomicrographs of L4 stage animals showing the direction of AVM axon protrusion from the cell body. Ventral is down and anterior is to the left. Scale bar: 20 µm. In the wild-type pattern, the AVM axon ventrally protrudes toward the ventral nerve cord (B). Loss of *unc-6* function causes anterior protrusion (C). Axon protrusion in the *unc-53* mutants is similar to that observed in wild-type animal. (D). In *unc-53(n152);unc-6(ev400)* double mutants, AVM axons frequently protrudes dorsally (E), posteriorly (F), or have short extra extensions (G). (H–M) Photomicrographs of L4 stage animals showing the direction of HSN axon protrusion form the cell body. Ventral is down and anterior is to the left. Arrow indicates the PLM axon. Scale bar: 10 µm. In the wild-type pattern, the HSN axon protrudes ventrally from the cell body. After reaching the ventral nerve chord the axon extends anteriorly and defasciculates from the cord to form synapses at the vulva (H). Loss of *unc-6* function causes anterior axon protrusion (I). Although most *unc-53(n152)* mutants have the wild-type protrusion pattern, in *unc-53(n152);unc-6(ev400)* mutants, HSN axons frequently protrude dorsally (J), posteriorly (K), or have extra extensions (L). Although most *unc-5(e53)* mutants have the wild-type protrusion pattern, in *unc-53(n152);unc-5(e53)* mutants, HSN axons frequently protrude anteriorly (M). PLM is not shown in J, K, L, or M because axons often terminate early in *unc-53(n152)* mutants.

We were interested in the role that UNC-53 might play in UNC-6 guidance. The AVM neuron projects an axon ventrally towards the source of UNC-6 and is often used to study UNC-6 guidance (Fig. 1A) (Chan et al., 1996; Hedgecock et al., 1990; Wadsworth et al., 1996). In mutants with the loss-of-function *unc-6(ev400)* mutation the AVM axon has a strong bias to migrate anteriorly, whereas mutants with the *unc-53(n152)* allele have the wild-type ventral projection. Surprising, a different pattern emerges in *unc-53(n152); unc-6(ev400)* double mutants; the axon will protrude in any direction; anterior, posterior, dorsal, or ventral (Fig. 1B–G; Table 1).

**Table 1.**
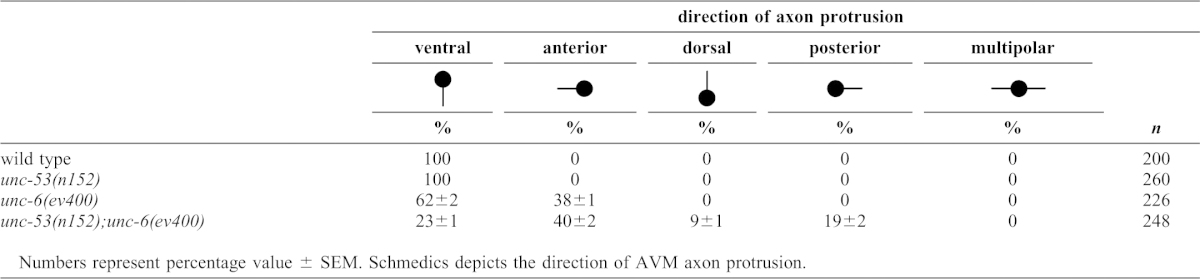
Direction of axon protrusion from the AVM cell body.

The unusual axon protrusion pattern observed from AVM, prompted us to examine the effects of *unc-53* mutation on axon protrusion from the HSN neuron. Similar to our observations of AVM, we also find a unique pattern of axon protrusion from the HSN neuron cell body in *unc-53(n152); unc-6(ev400)* mutants (Fig. 1H–L; Table 2). The axon protrudes from either the dorsal, anterior, or posterior sides of the neuron. This is similar to the phenotypes we previously observed in mutants with different point mutations in *unc-40* and *unc-6* (Xu et al., 2009). In that study, the *unc-40* point mutation, *unc-40(ur304)*, was shown not to disrupt axon protrusion unless in the background of the loss-of-function *unc-6(ev400)* mutation or the *unc-6(rh46)* mutation.

**Table 2.**
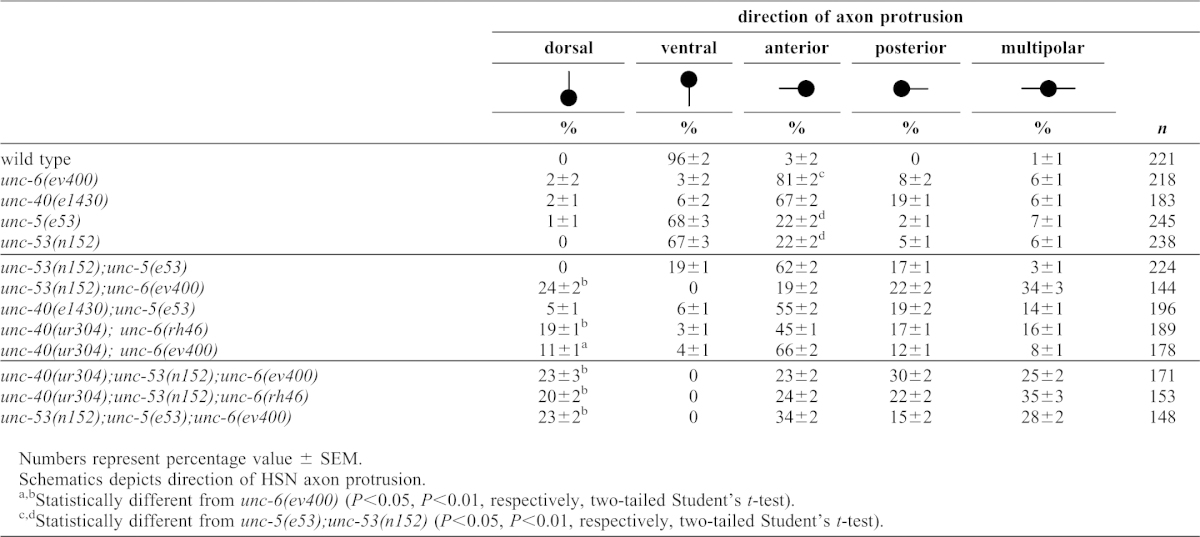
Direction of axon protrusion from the HSN cell body.

**Table 3.**
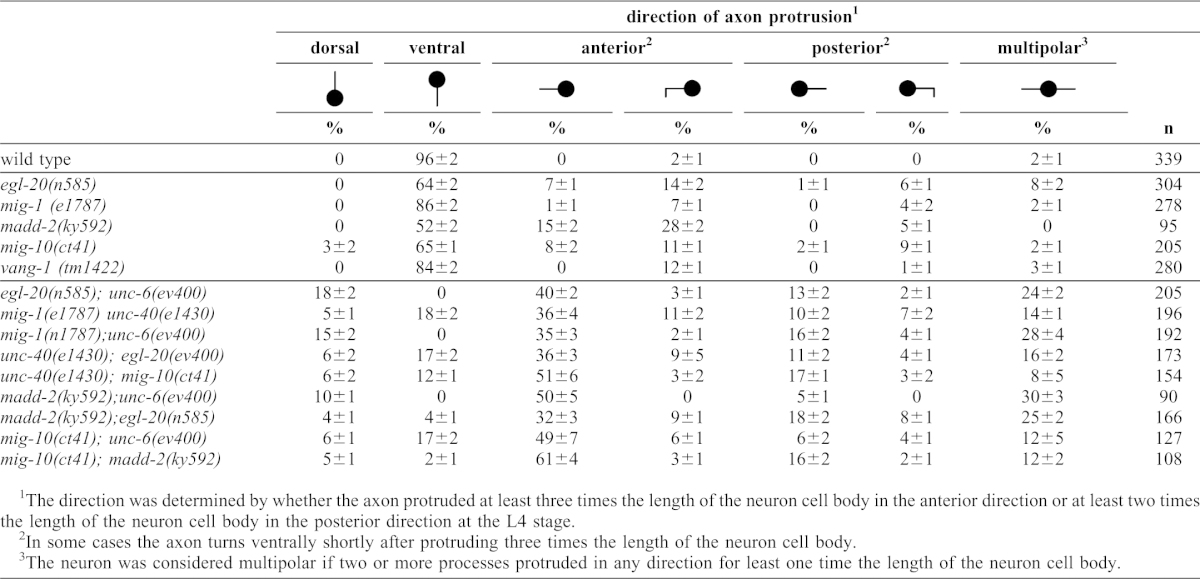
Direction of axon protrusion from the HSN cell body.

### UNC-40 localization is associated with the site of HSN axon outgrowth

UNC-40 is required for the UNC-6 guidance of the HSN axon to the ventral nerve cord (Hedgecock et al., 1990). There is strong evidence that the intracellular localization of UNC-40 is associated with the site of axon outgrowth. A GFP-tagged UNC-40 expressed in the HSN can rescue ventral HSN guidance in *unc-40* mutants and this rescuing UNC-40::GFP protein becomes localized to the ventral side of HSN beginning at the early L2 stage when a distinct ventral leading edge develops (Adler et al., 2006). Further, in animals expressing UNC-40 A1056V the direction of axon outgrowth is arbitrary in *unc-6* loss-of-function mutants (Xu et al., 2009). When expressed with UNC-40 A1056V in the *unc-6* mutant, the UNC-40::GFP reporter protein becomes localized randomly in one direction, suggesting that functional UNC-40 A1056V/UNC-40::GFP dimers are formed that can direct axon outgrowth in different directions (Xu et al., 2009).

**Fig. 2.**
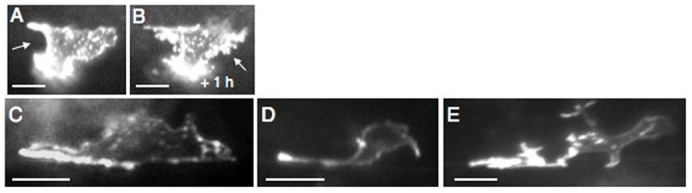
UNC-40::GFP is localized at the sites of axon outgrowth. Photomicrographs of the localization of UNC-40::GFP in the HSN neuron during the early stages of axon formation. UNC-40::GFP localization is associated with the sites of axon outgrowth. (A and B) An HSN neuron was imaged over a 1 h interval in the L2 stage. During this period a single broad leading edge forms ventrally. (A) Strong and uniform UNC-40::GFP fluorescence is observed on the left side (arrow) and at a leading ventral edge. (B) After 1 hour, strong and uniform UNC-40::GFP fluorescence is observed to the right (arrow), whereas the fluorescence on the left appears less uniform and intense. (C–E) During the L3 stage multiple neurites develop. Images show a progression of outgrowth. We note that the expression of UNC-40::GFP appears to exacerbate branching. See figure 1 in Adler et al. for a comparison to wild-type animals (Adler et al., 2006). Ventral is down and anterior is to the left. Scale bar: 5 µm.

We sought to further define the relationship between UNC-40 localization and axon outgrowth by performing time-lapse imaging on animals expressing UNC-40::GFP. During the L2 stage, when a single ventral leading edge forms over the course of several hours, UNC-40::GFP is ventrally localized. The UNC-40::GFP intensity across the domain can vary (Fig. 2A,B). At the mid-L3 stage, there is a surge of dynamic morphological changes as multiple neurites extend from the leading edge. One of these neurites extends to the ventral nerve cord, whereas the others disappear (Adler et al., 2006). While overall the neurites and their growth cones extend ventrally, the morphological changes are dynamic and extensions are observed in different directions. We observe that UNC-40::GFP is associated with the sites of axon outgrowth, regardless of the direction of the outgrowth (Fig. 2C–E). These results show that the morphological changes of the axon and the UNC-40 localization pattern are dynamic during axon formation. UNC-40 localization is associated with the site of axon outgrowth at all stages of the axon's development.

### Mutations affect the asymmetric localization of UNC-40

During a period of about 6 hours during the late L1 and L2 larval stages, the HSN becomes polarized as the ventral cytoplasm expands and develops into a leading edge. This is followed by a short ventral migration of the cell body and then the outgrowth of neurites that will lead to the formation of the axon (Adler et al., 2006).

**Fig. 3.**
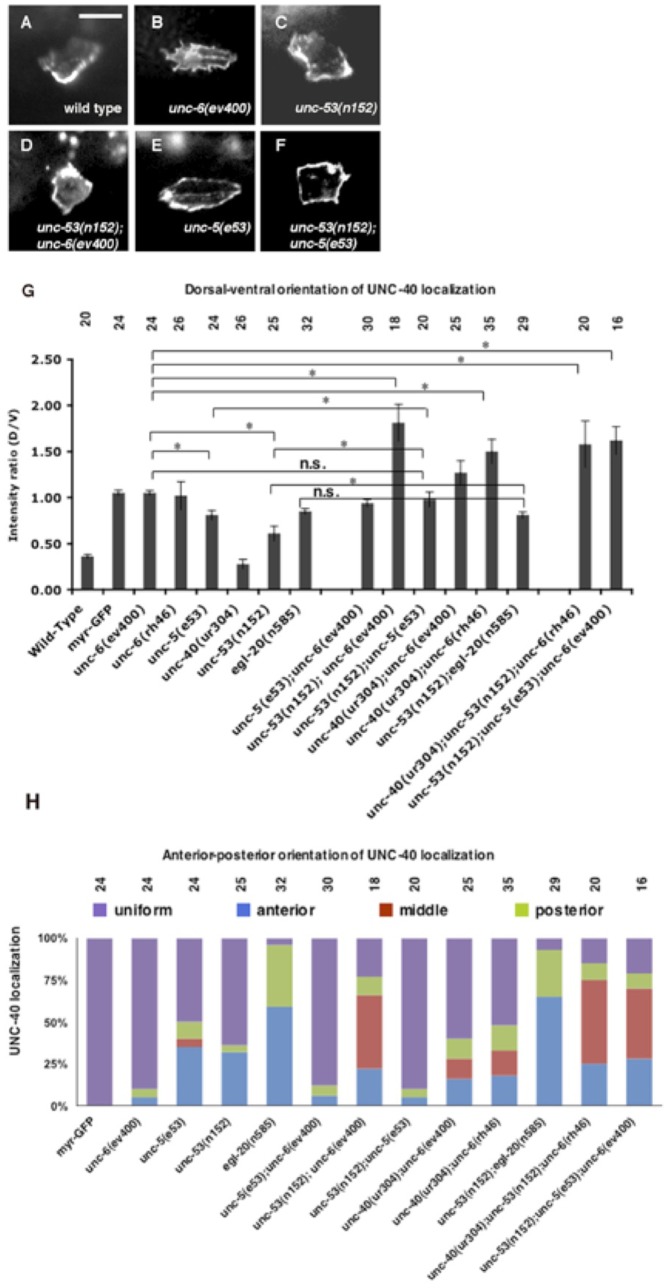
Genetic interactions between *unc-53* and *unc-6* and between *unc-53* and *unc-5* affect intracellular UNC-40::GFP localization. (A–H) Photomicrographs of the localization of UNC-40::GFP in the HSN neuron of L2 stage larvae. Ventral is down and anterior is to the left. Scale bar: 5 µm. UNC-40::GFP is ventrally localized in the wild-type animals (A), but in *unc-6(ev400)* mutants it is evenly distributed (B). Although UNC-40::GFP is ventralized in *unc-53(n152)* mutants (C), in *unc-53(n152); unc-6(ev400)* mutants it is polarized to different sides, including dorsally (D). Although UNC-40::GFP is ventralized in *unc-5(e53)* mutants (E), in *unc-53(n152); unc-5(e53)*mutants it is more evenly distributed (F) and is similar to localization in *unc-6(ev400)* mutants. (G) Graph indicating the dorsal–ventral orientation of UNC-40::GFP. The graph shows the average ratio of dorsal-to-ventral intensity from linescan intensity plots of the UNC-40::GFP signal around the periphery of the HSN cell. Wild-type animals show a strong ventral bias, whereas there is a uniform distribution in *unc-6(−)* mutants and an increased dorsal bias in *unc-53(n152); unc-6(ev400)* mutants. (*) statistic difference (*P*<0.05, one-tailed Student's *t*-test); (n.s.), statistically, no significance. (H) Graph indicating the anterior–posterior orientation of UNC-40::GFP. To determine orientation, line-scan intensity plots of the UNC-40::GFP signal across the dorsal periphery of the HSN cell were made, the dorsal surface was geometrically divided into three equal segments, and the total intensity of each was recorded. The effect of the mutations on UNC-40 distribution can be accessed by comparing the two graphs, for example in *unc-6(ev400)* mutants UNC-40 is evenly distributed as indicated by the equal dorsal-to-ventral ratio and the uniform anterior–posterior value. In contrast, UNC-40 is polarized to different sides in *unc-53(n152); unc-5(e53)*mutants as indicated by the increased dorsal-to-ventral ratio and the increased bias to localize at the middle of the dorsal side (red) or to the anterior (blue) or posterior (green) sides.

UNC-40::GFP becomes localized to the ventral side of the neuron beginning in the early L2 stage (Adler et al., 2006), whereas in *unc-6* loss-of-function mutants UNC-40::GFP is uniformly dispersed around the periphery of HSN (Fig. 3A,B,G,H). In the *unc-40(ur304)*;*unc-6(ev400)* or *unc-40(ur304);unc-6(rh46)* mutants intracellular UNC-40::GFP becomes localized in different directions (Xu et al., 2009). Because of the similarity of the axon protrusion phenotypes in *unc-40(ur304)*;*unc-6(ev400)* and *unc-53(n152);unc-6(ev400)* mutants, we examined UNC-40::GFP localization during HSN axon formation in the *unc-53(n152); unc-6(ev400)* mutants. In these experiments, we first assay for the distribution of UNC-40::GFP along the dorsal–ventral axis (Fig. 3G). In the mutants there is a strong bias for UNC-40::GFP to become localized to the dorsal region. We then assay for any bias along the anterior–posterior axis, by examining the distribution along the dorsal side of the neuron (Fig. 3H). We find that UNC-40::GFP also becomes localized to the anterior and posterior sides of the neuron. The subcellular localization of UNC-40::GFP to different sides of the neuron is similar to phenotypes observed in the study using the *unc-40(ur304)* allele. Fig. 3H is a stacked column chart with each colored rectangle of a column indicating the probability of UNC-40 localizing in a direction within the time frame of the L2 stage when the single leading edge is forming. These results provide experimental evidence that the process of UNC-40 localization is a source of randomness, *i.e.* removing the UNC-6 bias reveals a stochastic fluctuation.

### Genetic interaction between *unc-53* and *unc-5*

UNC-6 is thought to interact with UNC-40 homodimer receptor complexes and with UNC-40 and UNC-5 complexes (Hong et al., 1999; Stein et al., 2001). We reasoned that UNC-5 might play a role in the induction of the polarized distribution of UNC-40. This function might not have been apparent in previous studies if the UNC-40 homodimer complex acts in parallel to the UNC-40/UNC-5 complex.

We find that although the *unc-53(n152)* mutation and an *unc-5* loss-of-function mutation both cause mild protrusion phenotypes, double *unc-53(n152); unc-5(e53)* mutants show a strong phenotype that is similar to *unc-6(ev400)* mutants (Fig. 1M; Table 2). More significantly, whereas a polarized distribution is induced in both *unc-53(n152)* and *unc-5(e53)* mutants, in the double mutants the induction of a polarized distribution of UNC-40 is defective (Fig. 3F–H). In the *unc-53(n152); unc-5(e53)* mutants UNC-40::GFP localization is similar to that observed in *unc-6(ev400)* or *unc-5(e53); unc-6(ev400)* mutants, i.e. UNC-40::GFP is not asymmetrically localized but is uniformly dispersed around the periphery of HSN (Fig. 3G,H).

**Fig. 4.**
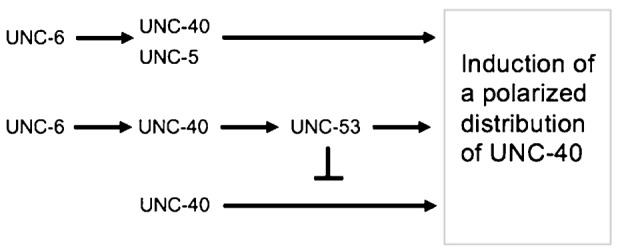
Genetic pathway. UNC-6-ligated UNC-40 induces a polarized distribution of UNC-40 in HSN (Xu et al., 2009). Genetic analyses indicate that UNC-6 induces UNC-40 polarization through parallel pathways comprising the cytoplasmic protein UNC-53 and the receptor UNC-5. The analyses also reveal an UNC-6-independent pathway that can induce the polarized UNC-40 distribution and which can be inhibited by UNC-53 activity.

Intriguingly, the double mutants *unc-53(n152); unc-5(e53)* and *unc-53(n152);unc-6(ev400)* have opposite UNC-40 localization phenotypes. It was, therefore, of interest to know whether either phenotype predominates in triple mutants. We find that the triple mutants, *unc-53(n152);unc-5(e53);unc-6(ev400)*, have axon protrusion phenotypes and UNC-40 localization phenotypes similar to the *unc-53(n152);unc-6(ev400)* mutants. That is, the HSN axon often protrudes from the dorsal or anterior sides of the neuron (Table 2) and there is also a strong bias for UNC-40 to become polarized to the dorsal region (Fig. 3G) and to the anterior and dorsal center sides of the neuron (Fig. 3H). These results indicate that UNC-40, independently of UNC-5, is capable of inducing UNC-40 polarization when not UNC-6-ligated and when UNC-53 activity is absent (Fig. 4).

We note that in these mutants the probability of UNC-40 localization and of axon outgrowth in the ventral direction is low. Our unpublished data suggest that a specialized basement membrane, which is just ventral of the HSN cell body, influences the probability of UNC-40 localization and of outgrowth in the ventral direction.

### EGL-20 (Wnt), MIG-1 (Frizzled) and VANG-1 (Van Gogh) restrict the site of UNC-40 asymmetric localization

**Fig. 5.**
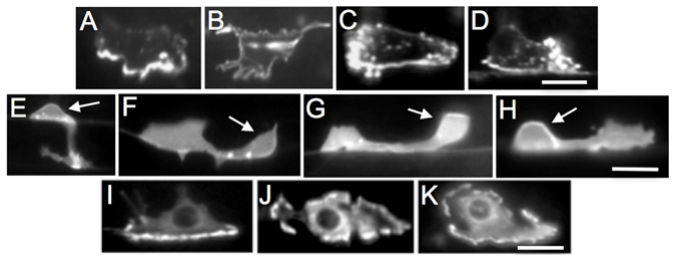
EGL-20 inhibits anterior and posterior orientation of UNC-40 asymmetric localization and the formation of axons from these sites. (A–D) HSN neurons expressing a functional UNC-40::GFP protein in the L3 larval stage. (E–H) HSN neurons in the L3–L4 larval transition stage expressing a marker to visualize the HSN neurons. Arrow points to HSN cell body. (I–K) HSN neurons expressing a functional MIG-10::GFP protein in the L3 larval stage. (A) In wild-type animals, the UNC-40::GFP protein is ventrally localized. (B) In *unc-6* mutants, the UNC-40::GFP protein is uniformly dispersed around the periphery. (C and D) In *egl-20* mutants, the UNC-40::GFP protein is localized ventrally as in wild-type animals or anteriorly (C) or posteriorly (D). (E) In wild-type animals the axon has a strong bias to ventrally protrude. (F) In *unc-6* mutants, the axon has a bias to form anteriorly. (G and H) In *egl-20* mutants the axon has a bias to form ventrally as in wild-type animals or anteriorly (G) or posteriorly (H). (I) In wild-type animals, the MIG-10::GFP protein is ventrally localized. (J,K) In *unc-6* and *egl-20* mutants, the MIG-10::GFP protein is uniformly dispersed around the periphery. Images are of collapsed stacks of optical sections. Ventral is down and anterior is to the left. Scale bar: 5 µm.

**Fig. 6.**
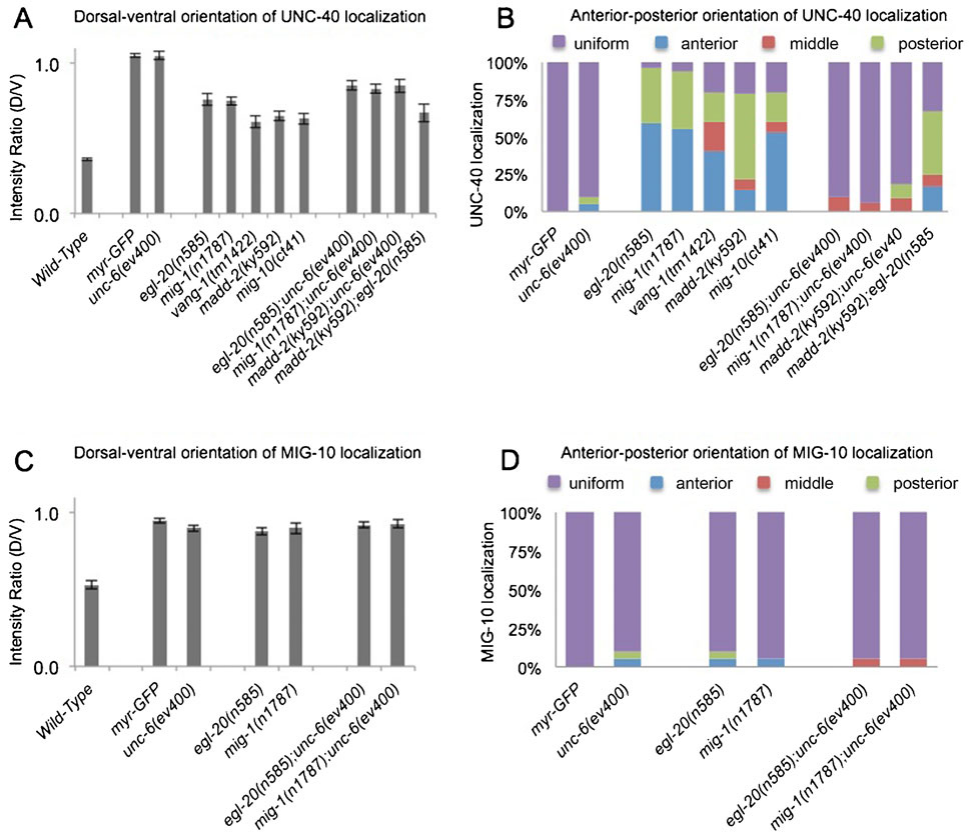
Genes function to inhibit anterior and posterior UNC-40 localization. (A) Graph indicating the dorsal–ventral orientation of UNC-40::GFP. The graph shows the average ratio of dorsal-to-ventral intensity from linescan intensity plots of the UNC-40::GFP signal around the periphery of the HSN cell. Wild-type animals show a strong ventral bias, whereas there is a uniform distribution in *unc-6(−)* mutants. In mutants there is an intermediate phenotype indicating a weak bias for ventral localization, as well as enrichment for localization at other sites compared to wild type (*P*<0.005, *t*-test). (B) Graph indicating the anterior–posterior orientation of UNC-40::GFP. To determine orientation, line-scan intensity plots of the UNC-40::GFP signal across the dorsal periphery of the HSN cell were taken, the dorsal surface was geometrically divided into three equal segments, and the total intensity of each was recorded. The percent intensity was calculated for each segment and ANOVA was used to determine if there is a significant difference between the three segments (see Materials and Methods). The measurements were taken using only the dorsal periphery in order to minimize cell shape differences. In several mutants there is a bias for anterior or posterior localization. (C) Graph indicating the dorsal–ventral orientation of MIG-10::GFP. The graph shows the average ratio of dorsal-to-ventral intensity from linescan intensity plots of the MIG-10::GFP signal around the periphery of the HSN cell. MIG-10 is ventrally localized in wild type, but the ratio is different in *unc-6(−)* and the mutants (*P*<0.005, *t*-test) because MIG-10 is uniformly distributed along the dorsal–ventral axis. (D) Graph indicating the anterior–posterior orientation of MIG-10::GFP. To determine orientation, line-scan intensity plots of the MIG-10::GFP signal across the dorsal periphery of the HSN cell were taken and analyzed as in Fig. 2B. There is a uniform distribution in *unc-6(−)* mutants and the mutants along the anterior–posterior axis. Error bars represent standard error of mean. *n*>15.

EGL-20 (Wnt) and the MIG-1 (frizzled) receptor affect the migration of the HSN cell along the anterior–posterior axis during embryogenesis (Desai et al., 1988; Pan et al., 2006). To determine whether *egl-20* and *mig-1* loss-of-function mutations affect UNC-40 localization in the anterior or posterior directions during HSN axon formation, we examine the localization of UNC-40::GFP in the mutants. While in wild-type animals the localization of UNC-40::GFP is ventral, in *unc-6* loss-of-function mutants UNC-40::GFP is equally distributed across HSN (Fig. 3A,B, Fig. 5A,B). The *egl-20* and *mig-1* mutations increase the dorsal/ventral ratio of UNC-40::GFP relative to wild type (Fig. 6A), indicating that UNC-40::GFP localization is also perturbed along the dorsal–ventral axis. However, unlike in wild-type animals or *unc-6* mutants, UNC-40::GFP is often asymmetrically localized to the anterior or posterior sides of HSN in *egl-20* and *mig-1* animals (Fig. 5C,D, Fig. 6B). Double mutants, *egl-20;unc-6* and *mig-1;unc-6*, have the *unc-6* phenotype; UNC-40::GFP is uniformly distributed (Fig. 6B). These results indicate that UNC-6 is required for UNC-40 to localize and they suggest EGL-20/MIG-1 signaling inhibits UNC-40 from localizing at the anterior and posterior sides of the neuron.

**Fig. 7.**
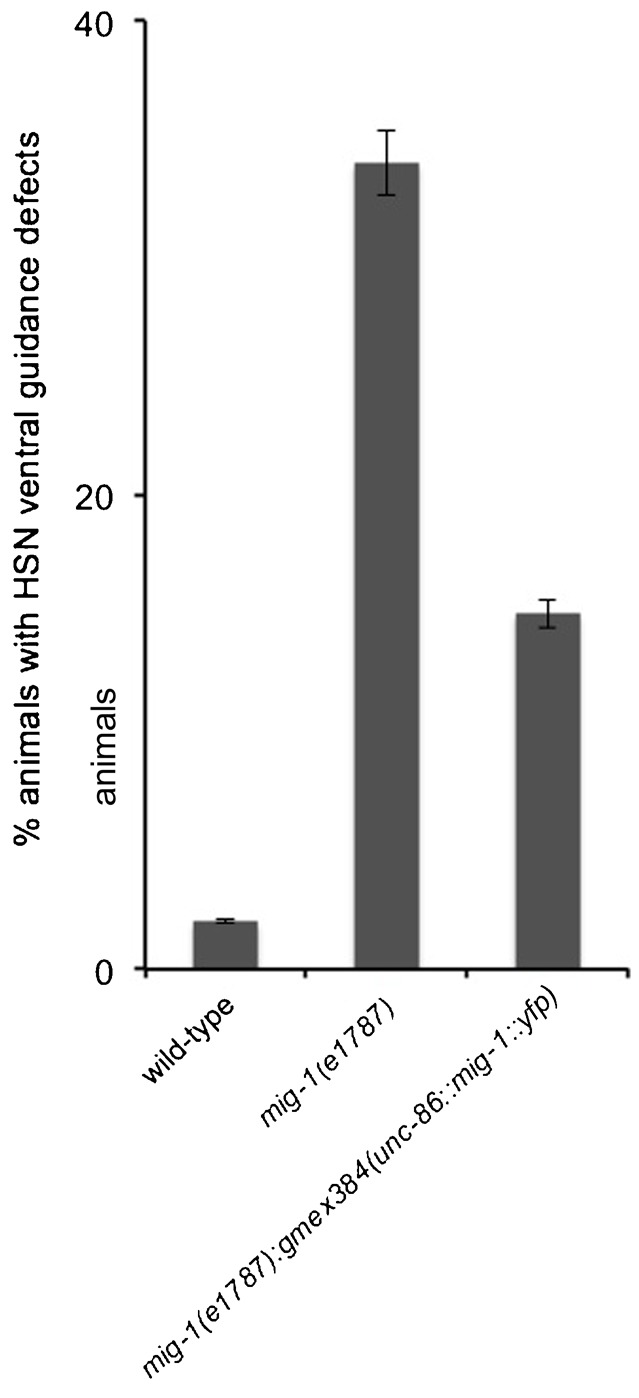
Cell-autonomous function of *mig-1*. The *gmex384(unc-86::mig-1::yfp)* transgene was introduced into the *mig-1(e1787)* genetic background. The *mig-1(e1787);gmex384(unc-86::mig-1::yfp)* animals exhibit partial suppression of the ventral guidance defect. “% animals with HSN ventral guidance defects” refers to the percentage of animals with any HSN axon that did not grow out towards the ventral midline.

A core component of the Wnt/PCP pathway is Van Gogh or Strabismus (Vang/Stbm or Vangl) (for a review, see Vladar et al., 2009). The only homolog of Van Gogh in *C. elegans* is encoded by *vang-1*. Similar to the phenotype of *egl-20* and *mig-1* mutants, in *vang-1* mutants UNC-40::GFP can be asymmetrically localized to the anterior or posterior sides of HSN (Fig. 6B). MIG-1 and VANG-1 most likely function within HSN since the MIG-1 receptor was shown to function cell autonomously to direct HSN migration in response to EGL-20 (Pan et al., 2006), and because VANG-1 has been shown to mediate a response to EGL-20 (Green et al., 2008) and is expressed in HSN (Sanchez-Alvarez et al., 2011). We further find that the expression of a *mig-1* transgene using a promoter to drive expression in HSN can partially rescue the HSN axon phenotypes of *mig-1(e1787)* (Fig. 7). Together these results suggest that EGL-20-VANG-1 PCP signaling inhibits UNC-40 from localizing in the anterior and posterior directions.

### UNC-40 effector proteins, MIG-10 and MADD-2, restrict the site of UNC-40 asymmetric localization

We hypothesize that EGL-20 Wnt/PCP signaling could organize cellular components that inhibits the ability of UNC-6-ligated UNC-40 signaling to cause UNC-40 localization at the anterior and posterior sides. UNC-40 is known to interact with CED-10/RAC-1 and MADD-2 (TRIM) proteins and it co-localizes with MIG-10 (lamellipodin), an actin-regulatory pleckstrin homology (PH) domain protein, at the ventral side of HSN (Adler et al., 2006; Chang et al., 2006; Hao et al., 2010; Krause et al., 2004; Quinn et al., 2006; Quinn et al., 2008; Song et al., 2011). We tested whether MIG-10 localization requires EGL-20 and MIG-1 and found that in *egl-20* and *mig-1* mutants MIG-10::GFP is uniformly dispersed around the periphery of HSN (Fig. 5J,K, Fig. 6C,D). The uniform distribution is also observed in *unc-6* and in *egl-20;unc-6* mutants. Together these results indicate that both UNC-6 and EGL-20 signaling are required to induce a polarized distribution of MIG-10 in HSN.

We find that the *mig-10* phenotypes are similar to that observed in *egl-20* and *mig-1* mutants, i.e. UNC-40::GFP is often asymmetrically localized to the anterior and posterior domains (Fig. 6B), as well as the ventral domain (Fig. 6A). MADD-2 can bind UNC-40 and it is also required for MIG-10 localization to the ventral side of HSN (Fig. 5I) (Alexander et al., 2010; Hao et al., 2010; Quinn et al., 2008). Consistent with a role for MADD-2 in MIG-10 localization, we find that the loss of *madd-2* also increases the probability that the polarized distribution of UNC-40 will orient to either the anterior or posterior side (Fig. 6B). These results indicate that similar to EGL-20 and MIG-1, MIG-10 and MADD-2 also plays a role in inhibiting UNC-40 from localizing at the anterior and posterior sides of HSN.

### Stochastic fluctuations of UNC-40 localization affects the timing of axon formation

**Fig. 8.**
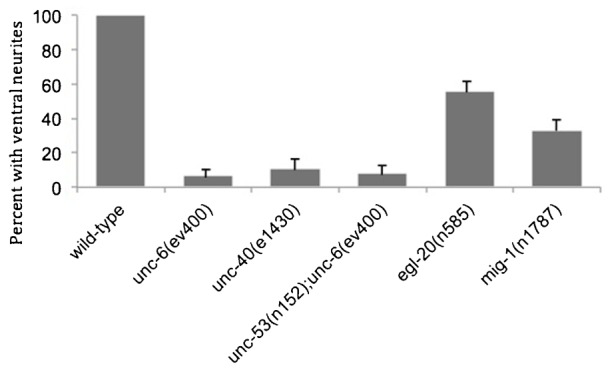
Timing of HSN axon development. The percentage of HSN neurons with predominantly ventral neurites in mid-L3. Whereas in wild-type animals there are ventral neurites, in the mutants there is a delay. In all strains an axon will form in the L4 stage. Error bars indicated the SEM; for each condition, *n*≧100 animals.

**Fig. 9.**
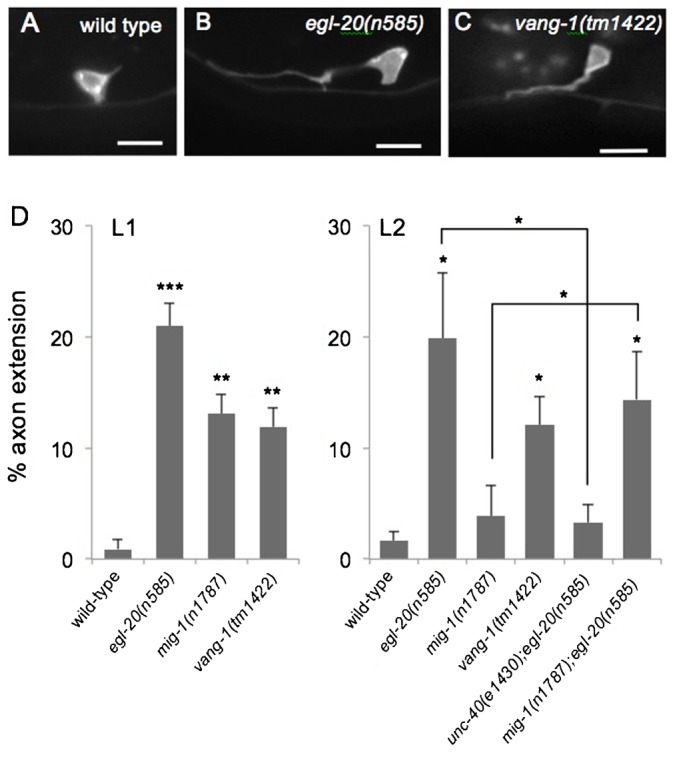
HSN axon extension in EGL-20 signaling mutants. (A–C) HSN axon extension at the L1 stage in wild-type, *egl-20(n585)*, and *vang-1(tm1422)* animals. Whereas in wild-type animals the axon protrudes in the early L4 stage, in the mutants axon protrusions are observed at earlier stages. Ventral is down and anterior is to the left. Scale bar: 5 µm. (D) Percentage of HSN neurons with a single axon in L1 and L2 stages. A neuron was scored as having an extension if the length of the neurite was at least twice the anterior to posterior length of the cell body. Significant differences (one-tailed Student's *t*-test), *P<0.05; **P<0.01; ***P<0.001; error bars indicated the SEM; for each condition, *n*≧105 animals.

In comparison to wild-type animals, the probability of UNC-40 localization at the ventral side decreases in the mutants, whereas the probability of localization at the anterior and posterior sides increases. Whereas there is little fluctuation in the direction of UNC-40 localization in wild-type animals, and much fluctuation in the UNC-40 (A1056V) and *unc-53;unc-6* experiments, in these mutants the fluctuation is intermediate with a ventral bias. Normally by the early L2 larval stage HSN is polarized ventrally with neurites primarily restricted to the ventral side where a leading edge forms; around the L3–L4 transition a single axon becomes evident (Adler et al., 2006). However in *unc-6* and *unc-40* mutants axon development is delayed and neurite extension is not confined to the ventral side during the L3 stage (Adler et al., 2006). We examined the morphology of HSN during larval development in *egl-20*, *mig-1*, and *vang-1* mutants and found that many of the neurons don't show clearly ventrally oriented neurites until around the L3–L4 transition (Fig. 8). In addition to there being fewer neurons with distinctly oriented protrusions at later stages, there is also some precocious axon protrusion. In *egl-20*, *mig-1*, and *vang-1* mutants there can be a distinct axon extension observed in the L1 and L2 stages (Fig. 9). In *unc-40(−);egl-20(−)* mutants the precocious axon extension phenotype is suppressed, suggesting that UNC-40 mediates the formation of the precocious axons.

## Discussion

Do the movements of axon guidance follow patterns that can be described quantitatively by statistical physics? The UNC-40 receptor binds the extracellular guidance cue UNC-6 and is required for axon outgrowth. In response to UNC-6, UNC-40 becomes asymmetrically localized and we show that there is a probability of UNC-40 being present at each of the different sides of the neuron during initial outgrowth. This provides experimental evidence for random fluctuations of UNC-40 activity within a neuron. We hypothesize the fluctuations of UNC-40 activity cause fluctuating sites of axon outgrowth and thus a succession of imbalanced outwards force across the surface of the neuron. The relationship between these stochastic fluctuations and the macroscopic properties of axon guidance can be described by non-equilibrium statistical physics. We propose that the observed behaviors of UNC-6 guidance approximate a random walk model. We experimentally tested this hypothesis using mutations that alter the fluctuations and we observe that the pattern of UNC-40 localization and axon outgrowth is consistent with this model.

### UNC-5 regulates the induction of UNC-40 asymmetric localization

A common model to explain UNC-6 axon guidance in *C. elegans* is that axons that express only the UNC-40 receptor are guided towards the ventral source of extracellular UNC-6, whereas those expressing the UNC-5 receptor are guided away. Thus the molecules and signaling pathways encoded by the neuron determine whether the axon has an attractive or repulsive response to UNC-6. Although this model explains most experimental results, there are some that are not consistent with this model. For example, the SDQR axon is directed away from the ventral source of UNC-6 in wild-type animals and is directed ventrally in *unc-6* null mutants, however the axon can be directed towards an ectopic dorsal source of UNC-6 in *unc-6* null mutants (Kim et al., 1999). Although the molecular properties of the neuron are presumably the same, the SDQR neuron seemingly has a different response to UNC-6 that depends only on the location of the UNC-6 source. In the present study, we find that intracellular UNC-40 in HSN can localize perpendicular to the axis of the UNC-6 source and axon outgrowth can be directed in the anterior and posterior directions in response to UNC-6. One simple interpretation is that the responses to UNC-6 are not exclusively attractive or repulsive.

We find that UNC-5 is required for the guidance of the HSN axon towards the UNC-6 source in the *unc-53* mutant. To our knowledge, this is the first report of an UNC-5 family member being required for attraction. Our results suggest that UNC-5 and UNC-53 act in parallel pathways to induce a polarized distribution of UNC-40 in HSN (Fig. 4). There is substantial evidence that the UNC-5 receptor is an UNC-6 receptor that cell-autonomously mediates signaling within a neuron for guidance (Hamelin et al., 1993; Hedgecock et al., 1990; Hong et al., 1999; Keleman and Dickson, 2001; Leung-Hagesteijn et al., 1992). The cytoplasmic protein UNC-53 is expressed in neurons and acts cell-autonomously for cell migration and axon guidance (Stringham et al., 2002). We find that UNC-40 is not asymmetrically localized in the *unc-53(n152); unc-5(e53)* mutants, suggesting that in *unc-53(n152)* mutants UNC-5 receptor activity is required to induce in HSN the signal that causes asymmetric UNC-40 localization. Although both *unc-5* and *unc-6* affect the guidance of cells and axons other than HSN, UNC-40 is asymmetrically localized in the *unc-53(n152);unc-5(e53);unc-6(ev400)* and the *unc-53(n152);unc-6(ev400)* mutants, indicating that the proper guidance of neighboring cells and axons is not required for UNC-40 asymmetrical localization in HSN. The simplest model is that UNC-5 functions cell-autonomously in HSN for the asymmetric localization of UNC-40 in *unc-53(n152)* mutants.

Rather than opposing roles in guidance, our results suggest that UNC-5 and UNC-40 act together and have distinct roles, *i.e.*, UNC-5 regulates the induction of UNC-40 asymmetric localization in HSN. Our observation that *unc-5* function can affect the guidance of neurons that primarily require *unc-40* function for guidance is a similar to earlier observations that the loss of *unc-40* function also affects guidance in neurons that primarily require *unc-5* function (Hedgecock et al., 1990). In fact, many neurons in both *C. elegans* and vertebrates are known to coexpress members of the UNC-5 and UNC-40 family of receptors (Chan et al., 1996; Leonardo et al., 1997). The concurrence of UNC-5 and UNC-40 signaling was also noted in ectopic *unc-5* expression studies, where it was concluded that the signaling mechanisms required for an UNC-5-mediated response to UNC-6 are present even in neurons that do not normally require UNC-5 activity for UNC-6 guidance (Hamelin et al., 1993). Together, these observations suggest a cooperative relationship between UNC-5 and UNC-40 signaling for axon guidance.

Because of the inconsistencies of our observations with the current model of attraction and repulsion, we wondered whether the results could be interpreted in a different manner. We previously showed that an UNC-40 variant, UNC-40 (A1056V) can induce the asymmetrical localization of UNC-40 in HSN even in the absence of UNC-6 and that the direction of the asymmetric localization in this case is indeterminate (Xu et al., 2009). This raised the possibility that the response of the HSN neuron to UNC-6 occurs through a stochastic process, and the neuron encodes neither an attractive nor repulsive response.

### UNC-40 activity randomly fluctuations

In this study, we show that wild-type UNC-40 can also be localized indeterminately in the *unc-53;unc-6* double mutants (Fig. 3H). Fig. 3H shows the results of observing the localization in multiple animals within the time interval of the L2 stage when the single leading edge is slowly forming (Fig. 3A–F, Fig. 5A–D). From this analysis we can derive a probability for UNC-40 localizing in each direction when an outgrowth is forming. For example, in *unc-53(n152);unc-6(ev400)* mutants there is a probability of 0.22 for anterior localization and a probability of 0.11 for posterior localization (Fig. 3H). The average dorsal-to-ventral ratio is high (1.8) indicating a higher probability for dorsal localization (0.67) than for ventral (0.36) (Fig. 3G). These results experimentally show that the direction of asymmetric localization of the UNC-40 receptor is randomly determined during an axon's outgrowth.

We observe by real-time imaging that fluctuations in the intensity of UNC-40::GFP fluorescence occur during the L2 stage (Fig. 2A,B). Using our imaging system we did not notice obvious fluctuations within the growth cones of the neuron at later stages (Fig. 2). However in theory, fluctuations of UNC-40 outgrowth activity could occur at the molecular level where the spatial and temporal differences could be difficult to directly observe. UNC-40::GFP may not cluster at sites of outgrowth. We are currently investigating different imagining techniques to study the dynamics of UNC-40 localization in the neuron.

While time-lapse imaging of UNC-40::GFP will provide a better understanding of the dynamics, it's the ability to derive the probability of UNC-40 being present at a specific side of the neuron during the L2 stage that provides the rational to probabilistically describe UNC-6 axon guidance. This is a new approach, as current research seeks to understand how the direction of UNC-6 guidance is determined by studying the molecular mechanisms of UNC-40 localization and axon outgrowth. A probabilistic approach considers axon guidance as a process that develops in time through the effects of random forces created by the fluctuation of UNC-40 outgrowth activity. Using this approach, the precise molecular mechanisms through which UNC-40 becomes localized and through which axon outgrowth takes place is less significant, as stochastic physics simply considers the mass effects that the outgrowth activity has on the movement of the axon at the macroscopic scale.

### Random walks model movement as a sequence of steps in random directions

The path that a randomly moving object takes is mathematically modeled as a random walk (Box 1) (Feller, 2008). The random walk is used to predict the diffusion of one gas into another, the spread of heat in a solid, and pressure fluctuations in a small container. The theory can be broadly applied, including to biological systems (Berg, 1993; Codling et al., 2008). For example, mathematical models have been used to predict that dynamic fluctuations of intracellular receptors in motile cells undergo a random walk. Membrane “patches” visualized in yeast and *Dictyostelium* cells have been shown to dynamically wander and have been mathematically modeled as undergoing a biased random walk (Dyer et al., 2013; Hecht et al., 2011). In the growth cone of neurons in culture, GABA receptors asymmetrically redistribute in the presence of a GABA gradient released by a pipette positioned perpendicularly to the axon axis. Mathematical modeling suggests that the kinetics of the relocalization is consistent with Brownian diffusion (Bouzigues et al., 2010).

The concept of a random walk was developed over a hundred years ago, primarily through attempts to understand Brownian motion. Brownian motion is the irregular motion of particles suspended in a fluid (Brown, 1828). The motion is caused by the instantaneous imbalance in the combined force exerted by collisions with the much smaller liquid molecules (which are in random thermal motion). In 1905, Einstein published a paper describing a model for Brownian motion (Einstein, 1906; Einstein, 1956). His approach became known as a “random walk” formalization because it considers the fluctuations of the molecular collisions as the cause of the motion (Duplantier, 2006; Frey and Kroy, 2005; Haw, 2002). One of his major insights was to use a probabilistic approach to obtain the *mean* displacement of a particle over time. Earlier attempts at modeling Brownian motion calculated the displacement of a particle by measuring velocity (the displacement divided by the observed time). However, the displacement of the particle is actually non-linear over time because of random motion. Einstein predicted that the mean displacement of the particle increases with the square root of time (Box 1). Einstein's predictions were experimentally tested and verified by Perrin. These experiments confirmed that the mathematical model could explain real-world observations of the movement.

A more phenomenological model to explain Brownian motion was presented by Langevin in 1908 (Langevin, 1908). This model simply considers the effect of the rapid collisions on a Brownian particle as a fluctuating random force (commonly referred to as the Langevin force). As this force changes with time, the position of the object the force is acting upon randomly changes. As a consequence, the path can be modeled as a random walk. The Langevin derivation is relevant to the hypothesis that the response of a neuron to a guidance cue is stochastic. In theory, random fluctuations of the site of axon outgrowth over time create a fluctuating force at the surface of the neuron, allowing positional changes to be modeled as a random walk (Box 1).

### A model of axon guidance by the stochastic fluctuations of UNC-40 outgrowth activity

Netrins cause axon outgrowth activity (Serafini et al., 1994). Therefore, they promote an internal force that causes movement. Our evidence suggests that the localization of the UNC-40 guidance cue receptor, which is required for directed axon outgrowth, can undergo stochastic fluctuations during the development of axon outgrowth. A biased random walk mathematically describes a path that consists of a succession of random steps where there is a consistent bias in a specific direction. This model describes how randomly directed steps over time can produce movement in a specific direction. In principle, the extracellular distribution of the UNC-6 guidance cue could introduce a directional bias into the stochastic fluctuations of UNC-40 outgrowth activity. It can be hypothesized that the extracellular distribution of UNC-6 creates such a strong bias for UNC-40 localization that normally there is very little fluctuation of the force driving axon outgrowth. In terms of the statistical mechanics, the system tends towards equilibrium where it does not have any preference for any of its available microstates. UNC-40 tends to localize only ventrally in wild-type animals and in this case the motion can be considered as straight-line, rather than being considered as a random walk. The stochastic nature of the axon guidance system comes into play when this bias is manipulated.

There are two predictions that can be made from a model in which UNC-40 outgrowth activity undergoes a biased random walk. These predictions are based on the probabilistic nature of the model. First, the direction of UNC-40 localization and axon outgrowth doesn't determine the direction of axon guidance. Rather guidance stems from the succession of random fluctuations of UNC-40 localization. The bias introduced by guidance cues at each step of the walk creates over time, on average, a “drift” in one direction. This means that at any discrete time, the direction of UNC-40 localization and axon outgrowth may not be towards the direction of guidance. Second, guidance cues affect how long it takes to form an axon. According to the model, guidance cues create a bias that affects the probability that UNC-40 will localize in a specific direction. If the bias is strong for only one direction, the displacement caused by the force takes a linear path. However, as Einstein realized, in a random walk the displacement grows only in proportion to the square root of time. Therefore, if manipulating guidance cues results in random fluctuations of UNC-40 outgrowth activity, the displacement of the axon will take longer on average to move away from the cell body.

### Experimental evidence that a random walk can model guidance

Our results show that the extracellular asymmetric distribution of UNC-6 is not the only factor that can regulate the direction of UNC-40 localization. UNC-40 localization at the anterior and posterior sides of the neuron is inhibited by the function of several different proteins. As the axon forms in the mutants, UNC-40 will localize to either the anterior, posterior, or ventral side of the neuron. The mutations alter the relative likelihood that UNC-40 will be present at a specific location. The probability for ventral localization is higher than for dorsal localization. For example, in *egl-20(n585)* mutants there is a probability of 0.59 for anterior localization and a probability of 0.37 for posterior localization (Fig. 6B). The average dorsal-to-ventral ratio is low (0.75) indicating a higher probability for ventral localization (0.57) than for dorsal (0.43) (Fig. 6A). (Presumably the bias for ventral localization increases as the outgrowth moves towards the ventral source of UNC-6.) This provides the means to experimentally test whether a biased random walk model can describe the behavior of UNC-6 axon guidance.

Remarkably, the behavior of this complex biological system approximates a biased random walk model. It is observed that in the mutants the HSN axon will be guided ventrally even though the initial UNC-40 localization and axon outgrowth can be anteriorly or posteriorly directed. As described by a biased random walk, the direction of UNC-40 localization at any discrete time doesn't predict the direction of axon guidance. Rather, the direction of guidance is caused by the successive stochastic fluctuations of UNC-40, which on average over time directs outgrowth towards the source of UNC-6. The mutations also cause axon formation to be significantly delayed in comparison to wild-type animals. This can be explained by the effect that random fluctuations have on the average displacement. Formally, the mean square displacement (msd) tends to increase only linearly with time in the mutants, whereas the msd tends to increase quadratically with time in wild-type animals, where random fluctuations are reduced. In other words, because of the stochastic fluctuations in direction, the axon takes longer to grow out in the mutants. In wild-type animals, the random fluctuations are reduced and the force is directed in a straight line, where distance becomes proportional to the time interval (distance equals velocity × time). Finally, while UNC-6-ligated UNC-40 induces asymmetric UNC-40 localization, Wnt/PCP signaling is inhibitory. A lack of Wnt/PCP signaling early in the guidance process increases the probability that the outgrowth process will begin too soon.

## Conclusion

Our evidence indicates that the response to the UNC-6 guidance cue is stochastic. A process is induced that causes the localization of the UNC-40 receptor and axon outgrowth activity in a random direction. This stochastic fluctuation can be repressed by the effects of the extracellular UNC-6 gradient and by the function of several genes. Together these factors create a strong bias for UNC-40 localization and axon outgrowth activity in only one specific direction. However, when this bias is altered, UNC-40 activity stochastically fluctuates and there is a probability for UNC-40 outgrowth activity at each side of the neuron. The movement of the axon than approximates a random walk.

In a deterministic model, the intracellular localization of UNC-40 towards the source of UNC-6 determines the direction of HSN axon guidance. It could be argued that *in principle* HSN axon guidance must be deterministic. After all, if the effects of UNC-40 outgrowth activity were known at each discrete time during outgrowth, the direction of axon guidance could be precisely determined. However, using the probabilistic approach taken here, we conclude that the random fluctuation of intracellular UNC-40 outgrowth activity determines the direction of HSN axon guidance. We observe in the mutants that at any discrete time UNC-40 and axon outgrowth can be directed anteriorly or posteriorly, although over time the axon is guided ventrally. The movement of any object (whether it is a Brownian particle, a forging animal, or a stock price) that is described as a random walk has this property. That is, whatever causes the object to move in a specific direction at one particular time doesn't determine the overall trajectory of the object over time. This raises an unintuitive and controversial conclusion that the molecular mechanisms that regulate the direction of axon outgrowth don't determine the direction of axon guidance. In this model, guidance molecules regulate different aspects of the stochastic process. Some, such as UNC-5 and UNC-53, regulate the induction of the stochastic process, whereas others, such as EGL-20 and MIG-10, create the bias that provides specific directionality.

It is increasingly recognized that random fluctuations are fundamentally important to many natural phenomena. They influence the behavior of complex systems, including cells, organisms, ecology, and the atmosphere. The results presented here suggest that the stochastic fluctuation of UNC-40 outgrowth activity underlies the ability of the neuron to determine the direction of guidance in response to UNC-6. This hypothesis implies that axon guidance is a biological process that makes and exploits random fluctuations to create function.

**Box 1. Random walk model.**

*Left*: A schematic of a random walk of *N* steps that ends at distance *X*.

*Right*: Illustration of a hypothetical random walk caused by the stochastic fluctuation of intracellular UNC-40 receptor localization. Sites where axon outgrowth is directed (red dots) randomly fluctuate over time causing a succession of outgrowths (shades of gray).

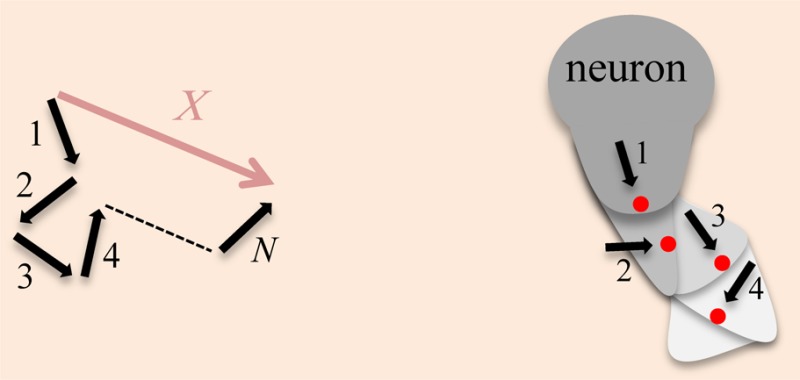

A random walk model can predict the average distance traveled in a walk of *N* steps. Here is a simple model for a walk in one dimension to illustrate formulization of movement. The same basic model can be applied to walks in two or three dimensions, and can be extended with more sophisticated methods of including different probabilities and unequal steps.

Envision a ‘‘walker’’ at each time period Δ*t* deciding to take one step of length *δ* to the right or left at random. With each step the walkers location changes by Δ*x* = ± *δ*. After *N* steps the walker's location will be:
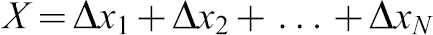
However, a walker's location can vary between +*Nδ* and −*Nδ*. Therefore, the average distance over a large number of different walks, all with *N* steps, will be zero because the probability of a right or left direction is equal. Since *X*^2^ is always positive, the square root of that average can be used to find the average distance from the origin of the walk after *N* steps:
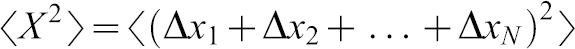
The sums in this equation are just the length of the corresponding step in the random walk, so that 
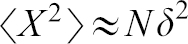
. Taking the square root of both sides of the equation obtains the expression for the root mean square:
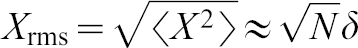
This is an important result since it means that the distance in a random process increases only in proportion to the square root of the number of steps, or, in other words, by the square root of the time.

The difference in the characteristic distance covered by an object undergoing a random walk versus one with straight-line motion can be significant. For example after a million steps of one millimeter in length:
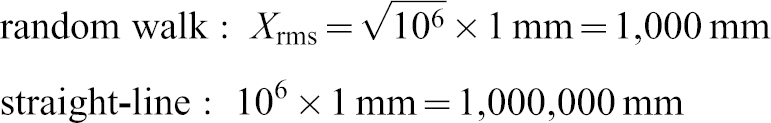
The mean square displacement (msd) is a measure of the average distance a molecule travels. With motion described as a random walk the msd increases only linearly with time. If random movement is not present, movement is effectively in a straight line away from the origin and the msd increases quadratically with time.
